# "It gets people through the door": a qualitative case study of the use of incentives in the care of people at risk or living with HIV in British Columbia, Canada

**DOI:** 10.1186/s12910-020-00548-5

**Published:** 2020-10-27

**Authors:** Marilou Gagnon, Adrian Guta, Ross Upshur, Stuart J. Murray, Vicky Bungay

**Affiliations:** 1 Canadian Institute for Substance Use Research, University of Victoria, 2300 McKenzie Ave, Victoria, BC V8N 5M8 Canada; 2 School of Social Work, University of Windsor, 167 Ferry Street, Windsor, ON N9A 0C5 Canada; 3 Dalla Lana Chair in Clinical Public Health, Dalla Lana School of Public Health, 678-155 College Street, Toronto, ON M5T 3M7 Canada; 4 Canada Research Chair in Rhetoric and Ethics, Department of English Language and Literature, Carleton University, 1125 Colonel By Drive, Ottawa, ON K1S 5B6 Canada; 5 Canada Research Chair in Gender, Equity and Community Engagement, School of Nursing, University of British Columbia, T201-2211 Wesbrook Mall, Vancouver, BC V6T2B5 Canada

**Keywords:** AIDS, Canada, Behavioural economics, Case study, Ethics, HIV, Incentives, Qualitative

## Abstract

**Background:**

There has been growing interest in the use of incentives to increase the uptake of health-related behaviours and achieve desired health outcomes at the individual and population level. However, the use of incentives remains controversial for ethical reasons. An area in which incentives have been not only proposed but used is HIV prevention, testing, treatment and care—each one representing an interconnecting step in the "HIV Cascade."

**Methods:**

The main objective of this qualitative case study was to document the experiences of health care and service providers tasked with administrating incentivized HIV testing, treatment, and care in British Columbia, Canada. A second objective was to explore the ethical and professional tensions that arise from the use of incentives as well as strategies used by providers to mitigate them. We conducted interviews with 25 providers and 6 key informants, which were analyzed using applied thematic analysis. We also collected documents and took field notes.

**Results:**

Our findings suggest that incentives target populations believed to pose the most risk to public health. As such, incentives are primarily used to close the gaps in the HIV Cascade by getting the "right populations" to test, start treatment, stay on treatment, and, most importantly, achieve (and sustain) viral suppression. Participants considered that incentives work because they "bring people through the door." However, they believed the effectiveness of incentives to be superficial, short-lived and one-dimensional—thus, failing to address underlying structural barriers to care and structural determinants of health. They also raised concerns about the unintended consequences of incentives and the strains they may put on the therapeutic relationship. They had developed strategies to mitigate the ensuing ethical and professional tensions and to make their work feel relational rather than transactional.

**Conclusions:**

We identify an urgent need to problematize the use of incentives as a part of the "HIV Cascade" agenda and interrogate the ethics of engaging in this practice from the perspective of health care and service providers. More broadly, we question the introduction of market logic into the realm of health care—an area of life previously not subject to monetary exchanges.

## Background

There has been growing interest in using incentives to increase the uptake of health-related behaviours and achieve desired health outcomes at the individual and population level [1]. The belief that incentives can help address complex (read expensive) health issues is rooted in behavioural economics. This field posits that incentives can increase the perceived reward of health-related behaviours and, in turn, increase the likelihood that people will engage in such behaviours [2]. However, the use of incentives remains controversial for ethical reasons. Ethical concerns include but are not limited to free and informed consent, autonomy and decision-making, privacy, vulnerability, the potential risk for undue inducement and coercion, and changes to the therapeutic relationship [3]. An area in which incentives have been not only proposed but used is HIV prevention, testing, treatment and care—each one representing an interconnecting step in the "HIV Cascade" [4, 5]. Incentives have been advanced in HIV following the adoption of global targets to achieve "90–90–90" (90% of all people living with HIV tested, 90% of all people diagnosed taking antiretroviral treatment, and 90% of all people on treatment achieving viral suppression [6, 7].

There are two applied categories of health-related incentives: positive and negative. Positive incentives reward and reinforce the desired behaviour or the achievement of a specific outcome [8]. For example, receiving a $20 monthly cash incentive to complete a Hepatitis B immunization program [8]. Positive incentives can take the form of cash payments, prizes, vouchers, gift cards, a chance to participate in a draw, and other rewards such as food, clothing, or personal hygiene products. They have been used in various clinical areas to encourage clinic attendance, follow-ups, treatment adherence, immunization, screening, smoking cessation, weight loss or maintenance, healthy eating, exercise, substance use reduction or abstinence, and breastfeeding [8–21]. Negative incentives involve withdrawing a potential reward when an individual fails to adopt the desired behaviour or achieve a specific outcome [8]. For example, someone who takes part in an abstinence-based group program and whose urine sample turns out to be positive for drugs would not enter the weekly prize draw. Negative incentives can also involve extra financial costs intended to discourage unhealthy behaviours, such as raising taxes on cigarettes, alcohol, or high-calorie food and beverages [8].

The majority of research in this field has focused on the acceptability and effectiveness of using incentives for health-related behaviours. Two complementary systematic reviews on acceptability have been published to date, one compiling articles published before 2014 [14] and one for articles published between 2014 and 2018 [15] for a total of 128 studies conducted in settings comparable to our study setting, namely the US, the UK, Canada, Belgium, The Netherlands, New Zealand, and Australia. In the most recent review, the authors conclude that the acceptability of incentives remains "polarized" [15]. They go on to identify several factors that influence acceptability, including perceived fairness (i.e., who is targeted, who benefits, and who is potentially left out), impact on autonomy (i.e., hindering or maximizing autonomy), intervention design (i.e., type of incentives, length of intervention, messaging, and actors involved), and compatibility with equitable health care goals [14, 15]. It is important to note that acceptability studies have been primarily conducted with members of the 'general' public and health-related groups. Perspectives of health care and service providers made up only 18% of the literature (*n* = 23). Of the total literature reviewed, twelve articles focused exclusively on providers with only two articles specific to HIV providers and eleven combining perspectives of both providers and clients [14, 15]. While this literature is helpful to think critically about incentives in health care, it largely overlooks the professional and ethical tensions that arise when bringing incentives into clinical or community practice settings—especially when working with people who experience inequities in health care.

Concerning effectiveness, the literature published to date is unanimous that incentives can be effective at encouraging health-related behaviours and meeting target health outcomes—more effective than usual care [1, 20, 22, 23]. However, they do not yield long-term sustainable changes, which results in decreased effectiveness over time [1, 22, 23]. In the field of HIV, the evidence is mixed. For example, research demonstrates that incentives can be effective in encouraging one-time or periodic health-related behaviours such as HIV testing [24, 25]. However, incentives are not as effective for sustained, more complex and long-term, health-related behaviours such as condom use, treatment adherence, and engagement in care or sustained health outcomes such as viral suppression [24, 26]. The literature on incentives reflects the various steps of the HIV Cascade, namely prevention, testing, treatment, and care. The bulk of the literature on prevention focuses on cash transfer programs for youth and women, aimed at addressing structural risk factors (e.g., poverty and gender inequality), rewarding outcomes such as having a negative STI or HIV test or behaviours such as safer sex (e.g., condom use, number of partners, non-transactional sex) [24, 27–31]. There is also a body of literature on the use of incentives (e.g., cash, food and transportation vouchers, lottery, gifts) to increase uptake of male circumcision for HIV prevention [32]. For testing, incentives (e.g., cash, lottery, food vouchers) have been studied across various high-risk populations and in various settings, including clinical and non-clinical settings [24, 25, 33]. Finally, the literature on treatment and care, which is more recent and emerging, features interventions to optimize treatment adherence, linkage to care, retention in care, and viral suppression [24, 34–39].

The number of scholarly articles on the ethics of incentives in health care has been growing. Still, empirical data on the experience of health care and service providers using incentives in this context is lacking. In preparation for our study, we reviewed the theoretical literature on the ethics of using health-related incentives [2, 3, 40–53]. This literature draws on classic principles of health care ethics, and most commonly cites the following ethical issues:

- The grey area between a "nudge" (i.e., motivation) and a "shove" (i.e., coercion)
- The focus on people who experience the most inequities in health
- The implications for client autonomy and freedom of choice
- The transformative effects on client-provider relationships
- The infringements on privacy due to the need for reporting and monitoring
- The impact on intrinsic motivation, stigma, discrimination, and shame
- The attribution of responsibility on the individual as opposed to structures and systems
- The worsening or creation of health and social inequalities

Further empirical work is needed to understand how these issues arise in practice and how providers mitigate them. This work is particularly important in areas such as HIV, where incentives are increasingly being touted as the key to closing the gaps in the HIV Cascade and, in turn, achieving global 90–90–90 goals to "end the AIDS epidemic by 2030" [6].

The main objective of this study was to document the experiences of health care providers (e.g., physicians, nurses, social workers) and service providers (e.g., community-based workers, peer workers) with incentivized testing, treatment, and care in British Columbia, Canada. A second objective was to explore the ethical and professional tensions that arise from the use of incentives as well as strategies used by providers to mitigate them. The province of British Columbia (BC), Canada, was selected as an ideal case study for three main reasons. First, BC is one of three provinces that account for most people living with HIV in Canada, with close to 12,000 people at the end of 2016 [54]. Second, BC is the birthplace of HIV Cascade-specific initiatives, such as Treatment as Prevention®^1^ and the Seek and Treat for Optimal Prevention of HIV/AIDS program (henceforth STOP HIV/AIDS®).^2^ Third, BC has a long tradition of using incentives to reach people at risk or living with HIV dating as far back as a decade. For example, in July 2010, testing fairs were held in the Downtown Eastside (DTES), a Vancouver neighbourhood with considerably high rates of drug use, HIV, poverty, mental illness, and overdose [55]. Residents of the DTES received a $5 gift card and a free meal in exchange for completing a rapid HIV test on site [55]. These testing fairs paved the way for the normalization of incentivized HIV testing in British Columbia—and Canada more broadly. The extensive use of incentives in the field of HIV and the steady growth of incentivized HIV care in Canada, and more specifically in BC, provided a unique 'case' for our study. In this paper, we present our study findings and discuss what we can (and should) learn from the experiences of providers who work with incentives in HIV care.

## Methods

We conducted a qualitative case study as defined by Stake [56, 57], which offers a flexible approach to examining the complexities of a particular case in its real-life context. The use of incentives in the care of people at risk or living with HIV in BC provided an instrumental case to gain a deeper understanding of a broader phenomenon (i.e., incentivized health care). When selecting an instrumental case, it is important to ensure that the case is typical or representative of other cases; likely to lead to a level of understanding that is applicable beyond the boundaries of the case itself; and easily "accessible" (i.e., sites can be easily identified, informants can be reached and are willing to participate, documents can be accessed, etc.) [56]. The selected case for this study met all three criteria: it is typical of incentivized care, it is likely to lead a level of understanding that is applicable beyond HIV, and it is easily accessible. We decided to use Stake [56] because of the philosophical underpinnings of his approach. Stake's approach recognizes context and subjectivity as essential elements of understanding [58]. He also acknowledges the involvement of the researcher throughout the research process and the importance of using inductive, from the ground up, strategies to generate knowledge that is reflective of the context in which the study is being conducted, the range of perspectives captured during data collection, the interpretation(s) of the researcher(s) and the complexity of the issue(s) under investigation [58]. From this perspective, the goal of case study research is not to generalize from a single case but to produce an in-depth understanding of a carefully selected case that permits us to learn and think about a broader phenomenon [59].

Instrumental case studies draw on multiple sources of data [56, 57]. For this study, we included four sources of data: participant interviews, key informant interviews, documents, and field notes. We recruited health care providers (i.e., physicians, nurses, social workers) and service providers (i.e., community-based workers, peer workers) who work in not-for-profit community-based organizations, health centres, or HIV clinics or programs by circulating email invitations and recruitment e-cards. Participants were eligible to take part in this study if they: identified as a health care or service provider, had worked with people at risk or living with HIV in the past five years, and had a least one experience working with incentives. We conducted semi-structured in-depth interviews with twenty-five providers. We achieved data saturation after twenty interviews. However, we completed five additional interviews to confirm that we had indeed reached saturation. Interviews lasted, on average, 45–90 min and focused on the actual hands-on experience of working with incentives with particular emphasis on context, role, use, benefits, limitations, ethical tensions, and broader implications (see Table 1).

**Table 1:** Interview guide

Background context	1. Please describe your current role (a) What is your training and background? (b) What are your main responsibilities? (c) How long have you been working in this role? 2. What happens in a typical day for you? (a) Who do you see? (b) Who do you interact with?
Using incentives	1. Please tell me about your experience with incentives (a) What was the context (b) How were you involved in the process or implementation? (c) What were the overall objectives? 2. Please tell me about how incentives were used in the program (a) What was your role? (b) How were you supported in this role? 3. How did your colleagues respond to the use of incentives? (a) Can you share examples of positive and negative responses? 4. How did the patients/clients respond to the use of incentives? (a) Can you share examples of positive and negative responses? (b) Did any issue arise? If so which one and how were they addressed? 5. How did incentives impact your practice/service?
Incentives broadly	1. Based on your experience, what were the benefits of using incentives? 2. Based on your experience, what were the challenges of using incentives? 3. What role do you think incentives should have in health care? 4 How can we ensure incentives are provided equitably? 5. How can we best support health care and service providers? (a) What are the current gaps? (e.g., training, support, best practices) (b) How and who should we address these gaps?

We sent email invitations to nineteen key informants with expertise in professional practice, ethics, HIV, law, and policy. Six key informants agreed to participate and completed a semi-structured interview lasting, on average, 45–60 min. The other potential key informants either did not respond or declined, citing a lack of knowledge of incentives as the main reason. Key informant interviews focused on gathering background information on the use of incentives and, more specifically, on the history of this practice, the context in which it was implemented, the rationale for using it, the process used for implementing, evaluating, and supporting this practice, and frameworks (e.g., legal, professional, ethical) that may offer new insights into this practice and ethical tensions. These interviews allowed us to contextualize the use of incentives on a historical, political, professional, and theoretical level. During the interviews, key informants referred to additional documents to contextualize this practice further and inform our analysis (e.g., books, news stories, articles published in magazines or peer-reviewed journals, policy documents, reports, websites, written anecdotes, etc.). All interviews (participants and key informants) were audio-recorded and transcribed. Finally, extensive field notes were taken throughout the study to guide the data collection process, capture emerging ideas, identify areas that needed more clarity or background, inform the analysis, and create an audit trail. Field notes included but were not limited to key ideas discussed during interviews, recurring themes, expressions used by participants, references to initiatives, programs, or projects with historical significance, contextual elements, analytical insights, and so forth.

Data analysis was primarily driven by participant interviews with other sources of data informing the process and acting as a necessary background for interpreting and situating the findings. To analyze the participant interviews, we used Applied Thematic Analysis (ATA) [60]. In summary, ATA involves four general steps: (1) read and code transcriptions, (2) identify possible themes, (3) compare and contrast themes, identifying structure among them, and (4) produce a thematic scheme to describe the research phenomenon. To complete the first step and develop a matrix, we analyzed six interviews that were selected based on data richness, diversity of experiences, and completeness (i.e., based on the interview guide). The matrix included six high-level themes, which emerged from the six interviews and reflected the interview guide, namely, (1) context, (2) goals, (3) incentives, (4) successes, (5) limitations, and (6) strategies. Then, the six interviews were analyzed by another member of the research team to ensure that the matrix developed throughout the first round was indeed complete. Those interviews were loaded into NVivo, which was used to complete the analysis process. Using the same matrix, the remaining interviews were analyzed in NVivo by a research assistant working under the supervision of the lead researcher. This matrix allowed us to work systematically through large amounts of data while keeping the focus of the analysis on specific content areas and identifying emerging themes. As the process evolved, we were able to refine the themes and identify sub-themes to develop a more nuanced, structured understanding of the findings.

## Results

We interviewed twenty-five providers, including nurses, social workers, community-based workers and peer workers (see Table 2). The majority of participants were nurses (*n* = 15). Out of the twenty-five participants, nineteen self-identified as women. The majority of them were in their thirties or forties. Two participants had completed a high school degree, six had completed a college degree, and seventeen had completed post-secondary education. More than 50% of the participants (*n* = 14) had been working in their current position between 5 and 10 years. Finally, 60% (*n* = 15) had been working with people at risk or living with HIV for the same amount of time.

**Table 2:** Sample characteristics

What year were you born?	
1950s–1960s	4
1970s–1980s	15
1990s	6
How would you describe yourself?	
Man	5
Woman	19
Non-binary	1
What is your highest level of education?	
High school	2
College	6
Undergraduate	16
Graduate	1
What is your position?	
Registered nurse	15
Social worker	2
Community worker	3
Peer worker	5
How long have you worked in this position?	
< 5 years	6
5–10 years	14
> 10 years	5
How long have you worked with people at risk or living with HIV?	
< 5 years	6
5–10 years	15
> 10 years	4

Interestingly, our sample also included perspectives of less experienced (less than five years) and more experienced providers (more than ten years). It is also worth noting that most participants had used incentives while working in various positions, including but not limited to their current position. As such, they were able to draw on a wealth of experience to explain the complexities of incentivized HIV care—and the tensions that arise from this practice. Four core themes emerged from the participant interviews: (1) Target populations and desired outcomes, (2) Perceived effectiveness, (3) Unintended consequences, and (4) Professional and ethical tensions. We present each theme in the sections that follow, but first, we want to set the stage for the findings and briefly describe some of the contextual factors extracted from both participant and key informant interviews.

### Context: situating our case study

We identified three contextual factors deemed to have played a significant role in the implementation and normalization of incentivized HIV care in BC, namely, (1) the launch and scale-up of STOP HIV/AIDS®, (2) the blurring of lines between research and clinical care, and (3) the transactional nature of survival micro-economies among the most marginalized groups of people at risk and living with HIV. As mentioned above, these factors were discussed in both participant and key informant interviews. They do not speak directly to the experience of using incentives. Still, they help locate this experience in a broader context and define our case—hence why we present it first and separately from the findings arising from the participant interviews.

The "birth" of incentives as we know them today in BC can be traced back to 2010 when the provincial government invested $48 million in scaling-up testing, treatment, and care via a three-year pilot project called STOP HIV/AIDS®. The goal of this program was to expand access to treatment as a tool for HIV prevention, with a particular focus on populations that are “hard-to-reach”, “hard-to-treat”, and “hard-to-retain” in care. In 2013, STOP HIV/AIDS® became a $20 million per year province-wide program that continues to be active today. This program has been credited for the steady decline in the number of new HIV cases and pioneering new approaches to care—including incentivized approaches. As one participant recalls, and as mentioned in the introduction, STOP HIV/AIDS® launched with a campaign of incentivizing HIV testing, particularly among hard-to-reach populations with high rates of undiagnosed HIV.

The STOP Program had just started, and part of that program was to get people tested in the Downtown Eastside. About 5000 people were tested down there over a 6 to 8-month period. The incentive for them was a $5 gift card. (Participant 7)

This campaign paved the way for incentivizing other areas of HIV care, such as treatment, retention in care, and treatment outcomes. It also paved the way for not-for-profit community-based organizations, health centres, and HIV clinics or programs across BC to take up incentives—albeit with more limited financial resources and not on the same scale as STOP HIV/AIDS®.

Against this backdrop, three phenomena overlapped and created the perfect conditions to move incentives from research to care. First, there was an intensification of HIV research in BC, which resulted in a constant flow of new opportunities to participate in studies and collect incentives. Second, studies that collected data for research purposes started using the same data for clinical purposes. An example shared by a few participants is one where a research participant providing a blood sample as part of a study would consent for results to be sent to their treating physician. Third, there had been a history of poor and marginalized people taking part in research as part of a survival economy. By the time incentives crossed over from research to care, those targeted were already familiar with the concept. One of our key informants summarized it as follows:

I think there was a precedent in a particular population that was targeted for this—like in the community, not so much in the clinic—but in the community. I mean, it was the same population who had been getting incentives for research projects for a while. So I mean, it was kind of out there in that regard before. So everybody was familiar with the concept around: "If you go and talk to this researcher, you'll get something." And then the testing fair thing was, you know, it really affected sort of the same population. (Key informant 3)

This quote leads us to our final contextual factor: the transactional nature of the micro-economies poor and marginalized people develop to survive. As many participants noted during interviews, 'time is money' when you live in poverty and need to hustle every day to take care of your basic needs. When faced with many competing priorities, decisions about health care may be relegated to the bottom of the list because they take considerable time (e.g., getting to a healthcare appointment and waiting to be seen). That is where incentives come in. If you need to get a blood test done, you may decide to go to the study site that gives you a $50 cash incentive for the blood test instead of your clinic, even if this contributes to potential delays or fragmentation in your care. Building on the same logic, if your clinic starts giving you a cash amount every day and a meal to take your medications, you may find time to go and take your medications. These examples, shared by participants, capture the transactional nature of survival micro-economies and reasons why such economies contribute to the success of incentives in these contexts. However, when incentives become the new standard of care, they can also make the care feel transactional and impact the therapeutic relationship.

I find it harder to build that rapport based on just, you know, getting to know someone and gaining their trust. It takes a lot longer with someone who is used to getting paid to do things or getting things for something, than say someone that has never experienced that (…). I find that a lot of people expect, you know, "Oh well, you know, where's my money?" I get that a lot, "Well, I'll go on meds if you pay me." And it's sort of like, "Well, I don't have any money." (Participant 1)

### Theme 1: Target populations and desired outcomes

Participants explained that incentives are not for everyone who faces barriers to accessing health care. Instead, they target specific populations based on their HIV risk. That is, people who are at risk of contracting HIV but are unlikely to test, diagnosed but not linked to care, linked to care but lost to follow-up, diagnosed but not on treatment, or treated but not fully adherent and suppressed.

*Participant*: Only people who come here are getting incentives. People who are getting tested at my regular clinic aren't getting incentives. So it's kind of, like is this fair? I don't know (…).

*Researcher*: So, why is this testing incentivized and not the other?

*Participant*: Probably because it's higher. Like the population we see is maybe a little bit higher-risk—or not maybe, is definitely a higher-risk (…). They could be infecting a bunch of people. (Participant 9)

These target populations are not only seen as having the most to gain through testing, treatment, and being retained in care; they also pose the most risk to public health, which is why they receive incentives, and others do not.

From a public health perspective, those are the people we need to be testing. Just in terms of protecting other people, you know, with transmission and infectivity. (Participant 10)

Participants saw incentives as deeply rooted in a particular public health logic. Still, they also saw some benefits in using them for a different (and more therapeutic) goal, namely, as tools for health promotion, engagement, and trust-building. One participant explained:

I believe the goal is to suppress people's viral loads, like to prevent infection to others. Also, to maintain people's health and wellbeing over the long-term (…). And have them engage with health care in some manner. And build trust and rapport with marginalized folks. (Participant 4)

As such, we identified a recurring tension between desired public health outcomes, which were top-down and measurable (e.g., number of tests done, number of new HIV cases found, number of pills missed, number of appointments attended, etc.), and those sought by participants, which were bottom-up and relational. Participants were aware that the primary goal of incentives was to "drive those numbers up," but they nonetheless tried using person-centred strategies. These strategies are discussed in the last section of the findings.

Participants had worked with a range of populations including adults and youth experiencing homelessness, gay, bisexual, and other men who have sex with men, people who use substances, people living with mental illness, sex workers, and undocumented migrant workers (including sex workers). They used incentives in two different ways. One, via brief, targeted initiatives designed to encourage participation in a group activity or uptake of HIV testing. Two, via medium- to long-term programs designed to optimize medication adherence and viral suppression, decrease substance use or maintain engagement in care. The following quotations illustrate the types of situations that would make someone eligible for one of those programs:

You have to be HIV-positive and very difficult to engage in a traditional care model. So let's say you usually don't follow through with your medication, you have unstable housing, or you live a relatively high-risk lifestyle. Or just basically, you're not engaging with the health care system. (Participant 11)

People are sick. Some people are quite sick. They have a ton of co-morbidities. And a lot of challenging relationships, or a lot of lifestyle factors that affect their ability to come to the clinic every day. So clients that are lost to care. They don't attend medical appointments. (Participant 13)

Clients who have fallen off taking their antiretroviral medications or their methadone. People that have no fixed address or homeless, so hard to locate. Yeah, lots of barriers to accessing health care, mental health, addiction. Just not wanting, not ready to, you know, go to a clinic and see a doctor. (Participant 17)

Individuals that the doctors are like, "You know what, they're having difficulty managing their medications. They're detectable, or they used to be undetectable." Or their partner is off antiretroviral medications. Like I think it usually is a few extra risk factors (…). And it will come to a team conversation, and everyone will agree that this person should get into the program. (Participant 18)

Participants described persons enrolled in incentive-based programs as typically struggling with multiple concurrent health conditions and street-entrenched. They lived busy, 'chaotic,' and complicated day-to-day lives with a clear focus on survival. Incentives worked to some extent because they brought hard-to-reach, hard-to-treat, and hard-to-retain populations through the door. However, as will be discussed in the next section, incentivizing is not a panacea when dealing with complex structural, social, and health issues. Incentives may drive numbers up, but according to participants, their effectiveness is superficial, short-lived, context-dependent, and one-dimensional.

### Theme 2: Perceived effectiveness

The vast majority of participants had used a range of positive incentives, alone or in combination, including a coin system ($1 or $2), cash amounts ranging from $5 to $50 (CAD), gift cards, draws (including contingency management fishbowl style^3^), food (snacks to full meals), candies, cigarettes, gifts, and clothing items. When asked why incentives work, participants were unanimous. They explained that incentives have the power to turn something that was not a priority into one, primarily (or exclusively) because a financial or material reward awaits and makes it worthwhile equivalent to the time, energy, and commitment. In the words of one participant:

Yeah. I mean, they definitely work. I think that there's a lot of people who would not get tested for various reasons if an incentive was not available to them. Some of those reasons are just priorities in their life: they're facing poverty, they're busy, they can't get tested. Or they to engage in other health care, and they need that $40 as well. (Participant 10)

In addition to this, participants explained that incentives work because they "break the ice" and send a signal to people who are living complicated lives that their time matters. This goes back to the idea that time is money, as previously mentioned, and taking care of one's health takes time.

It breaks the ice, I think. It motivates people, you know, to take a step or to engage in a program that they otherwise might not. So I think it's a bit of an ice-breaker in a sense. I believe that it can be, depending on the use or the context, really validating for a person in the sense that it acknowledges a valuing of their time and participation. (Participant 16)

Some participants identified poverty and hunger as the main reasons incentives work, that their effectiveness relies primarily on target populations desperately needing money (or gift cards that are more often than not exchanged for cash), goods, and food.

While participants agreed that incentives work, they considered their effectiveness superficial, short-lived, and one-dimensional. On the surface, the mere fact of incentivizing something when working with poor and marginalized populations increases your metrics of success (e.g., number of tests done, number of new HIV cases found, number of pills missed, number of appointments attended, etc.). However, as many participants pointed out, this is an overly simplistic way of addressing complex public health issues such as HIV prevention. Furthermore, this approach makes it easier to "gloss over those bigger problems and bigger needs" (Participant 10).

We're just like, "let's test and then move on." So I mean incentives, I understand incentives. It was very easy to understand. "Okay, we're getting more people tested, that's great." But then it's like, "What else are we doing?" (Participant 9)

This quotation captures a general concern shared by participants that incentives place too much emphasis on metrics rather than tackling existing structural barriers, social determinants of health (especially poverty and housing), gaps in knowledge, and failings of the health care system. The vast majority of participants also observed that incentives do not typically lead to profound and permanent changes in behaviours; instead, they tend to produce superficial changes that are short-lived. Once the incentives stop, the behaviours stop as well.

Many participants had seen incentives work to change behaviours and lead to desired outcomes in the short term, and then quickly stop working when incentives stopped (e.g., end of the intervention or program). Because of this, they considered the effectiveness of incentives to be short-lived and one-dimensional. This led to participants questioning if they are indeed the right tools to use (and fund) when working with people who struggle with treatment and care—and especially as we know from the beginning that they may not be financially sustainable over time. This participant summed it up:

I think if we had unlimited amounts of money in healthcare, then it would be great to have incentives. Just from the data that we took from it and experience, I don't think that it's worth it. I think we can utilize other engagement tools (…). I think in some cases, there's a place for incentives, but I also think that it can raise issues when they're taken away and people stop adhering to treatment or whatever they're being incentivized to do. So yeah, overall I would probably not choose to practise them. (Participant 5)

We identified three scenarios that capture the short-lived and one-dimensional nature of incentives. In the first scenario, an incentivized intervention is funded but only for a specific dollar amount and a limited amount of time. This intervention may be useful, but it stops producing results when the intervention stops, bringing you back to square one.

You know, because the money runs out or, you know, they realize that they don't want to take a medication or don't want to get their blood work done and they're still not going and doing it, so there's something else going on. (Participant 1)

In the second scenario, an incentivized intervention is offered overtime, but reaches a plateau effect and ceases to produce the type of effectiveness noted initially—pointing to more complex and deeply-rooted issues that cannot be solved by incentives alone.

Initially, there is a really powerful effect of incentives. But yeah, it can get to the point where if a person's getting the same incentive time and time again, that there is, the value of it decreases, I think. That it becomes a less, less sought-after reward, less exceptional, more normal and, therefore, less impactful. (Participant 16)

In the third scenario, an incentivized intervention designed to boost the perceived reward of engaging in treatment and care becomes deceiving or normalized over time; thus, it is no longer seen as valuable, worthy of time or energy. To sum up, participants recognized that incentives could be useful but only as a "Band-Aid solution." They did not solve the more significant issues, nor did they produce deeper, long-lasting, or all-encompassing behavioural changes.

I think incentives are like a bandage solution. I find that it's effective, it's useful, but it doesn't really solve the key problems, especially among people I work with. Because the two key things are lack of stable housing, and the chaotic lifestyle associated with substance use. So I guess my point is incentives are very useful as a band-aid solution but not solving bigger problems in society. (Participant 11)

### Theme 3: Unintended consequences

When they are implemented in the context of health care or service provision, incentives can have unintended consequences. Participants felt that they had little space to talk about these consequences in practice, and they typically dealt with them to the best of their ability, on a case-by-case basis and using strategies they had learned in practice. Because they were tasked with administering incentives, but not necessarily included in decisions that shaped how and which incentives were used, their insights were extremely valuable to understand issues that may arise at the point of care. A good example of this is the preferred use of gift cards over cash incentives.

To be honest, our hands were forced. I would have rather given just cash. People would tell stories about how we'd provide them with these gift cards, and then they'd have to go out and find someone to buy them off of them because they didn't actually want to shop at that store. They wanted to use the money in some other ways. So it would have been preferable to give cash. (Participant 16)

From an administrative, accountability, and liability standpoint, gift cards may make perfect sense. They are easy to account for, easy to buy, easy to manage, and so forth. They also direct people to spend money on items deemed acceptable and essential such as food and clothing. However, in reality, gift cards can be problematic. In addition to being paternalistic and restrictive, as pointed out by participants, they may force someone into a space where they do not feel safe or force them to resell at street value (i.e., half of the card amount). As such, they put participants in a difficult position.

Gift cards just suck, because a lot of times clients get gift cards to like [name of a chain store] and it's like, you can't really buy anything for that amount. It's like you can buy a meal and probably a coffee. And you can't really go and sell that downtown. And also, if it's a place that you don't want to go to, like [name of a chain store]. It's so frustrating because it's like we're telling them to go there and it's a really uncomfortable place to go. (Participant 20)

For these reasons, participants found that gift cards were not ideal. However, they did mention that gift cards provided a sense of safety because dealing with cash incentives could pose a risk.

I'm alone in that room. I find there are often security issues on their own just being in the space I use—that's a whole other story. But, so I don't, I don't know if I feel comfortable having like a lot of cash and then handing it out. (Participant 23)

Participants raised safety concerns for themselves and people who were on the receiving end of cash incentives. For example, when places that give out incentives became known to the community, it could put people at risk of being targeted—especially if they received cash. As one participant recalled: "I've taken people out back doors. You know, because somebody is waiting for them, because either they have a drug debt or a controlling partner" (Participant 26).

Throughout the interviews, we not only learned that incentives could have unintended consequences on the safety of providers and those who receive them, but we also found that incentives could inadvertently contribute to a sense of unfairness among the broader community. In other words, distributing incentives unevenly among populations of people at risk or living with HIV could be perceived as unfair. This situation created two significant challenges. First, participants had to engage in the process of triaging to ensure eligibility while trying to skillfully justify why this triaging was necessary and redirecting people who were not eligible to another service or resource.

We have had people who have shown up at the group, and we have actually had to ask them or let them know that they're not eligible (…). So it's sort of a triaging type process that happens. Yeah, it's a bit of an artful one, especially if it's in a mixed kind of community where, for example, some people are HIV positive, but not everybody is. And those who are positive are getting access [to the incentives] (…). Usually, people [who are not eligible] are really understanding (…). Some people will be, you know, will, or have argued their case (…). And again, my goal would be helping them connect with another resource. (Participant 16)

Second, participants had to make sure that people who felt excluded, let down or treated unfairly did not put themselves at risk to gain access to incentives. In other words, they had to make sure that incentives were not sending "the wrong message" by linking heightened HIV risk with greater access to resources and support:

A lot of folks in the Downtown Eastside or partners of people living with HIV want to try to get in. They'll say, "I should just contract HIV, at least I get, you know, money and housing and better food and this and that." And that's actually, you see people doing that, and it's really sad. (Participant 1)

I've actually had people say, "What do you gotta do to get this type of service? Do I gotta get myself HIV?" And I was like [*gasp*]. It's like a huge system flaw, I think. I just think that we don't put obviously enough, there just, there just isn't enough for the people with addiction and mental health issues. (Participant 21)

Participants explained that they had to be very careful when talking to people who did not fit the eligibility criteria to ensure they were not incentivizing the "wrong thing." For example, when people who struggle with treatment and have a detectable viral load receive incentives, it may have the unintended effect of encouraging people who would otherwise not be eligible to get off their medications and become detectable—and then access incentives.

I had to be very careful with what I was saying, because of that. I was very concerned. So I was very careful with how I kind of explained everything. But yeah, I had some people saying, like, "Oh, your viral load is 89. We can't take you." They're like, "Oh, well, I'll come back next week." (Participant 4)

Because of this, some participants felt that incentives could be counterproductive, especially when the needs for support and care are so great across the community.

Finally, participants highlighted that incentives could have unintended consequences on relationships and engagement in care. From a relational perspective, it was not always easy for them to be in a position of having to administer incentives. It made the relationship feel more "transactional," as explained by this participant:

Of course, money changes everything. Money always changes everything, even if it's five dollars; it changes things, you know? And so, I don't know that it would be holistic or, you know, person-centred to say, okay, "If you do this, I'm going to give you $5." It could change the dynamic in that way where you are the gateway to something that they don't have enough of because they're completely marginalized (…). It could end up being damaging to the relationship. (Participant 8)

It also reinforced power dynamics by positioning the participants as the "holder of incentives"—the person who decides who gets incentives, how much they receive, if and when incentives are taken away or stopped, when rules can or cannot be bent, and so forth. It was evident in our analysis that participants exercised enormous power and flexibility in the actual administration of incentives. To quote one participant: "I mean literally the buck stops with me. I do have the power or the ability to bend the rules, or to sweeten the deal or whatever it might be" (Participant 16). Power dynamics were front and centre as participants described their experiences.

Being a health care provider, you just naturally have power in that. Like there is an undeniable power dynamic, especially if there's any kind of sense that you're holding something that someone wants and you have the ability to take it away. So, and that's a hard thing to name in a really gracious and graceful way. And I haven't met a lot of people who are really, really great at that. (Participant 25)

Because incentives could intensify power dynamics and make the relationship feel more transactional, they could also have some unintended consequences on engagement in care. Participants expressed two main concerns: (1) fragmentation resulting from clients organizing their care based on which clinics, organizations, and programs are giving out incentives, and (2) disengagement from areas of care not incentivized. Overall, the use of incentives did not always align well with the participants' actual role as service and care providers. As such, it raised some professional and ethical tensions, which are detailed next.

### Theme 4: Professional and ethical tensions

Participants described how incentives created new responsibilities in addition to their regular work, which was already challenging. They were now responsible for managing three things. First, they had to manage incentives. Depending on the incentives used, this could mean anything from handling money or gift cards, carrying cigarettes, buying people food or coffee, or supervising draws and giving out rewards. In the absence of best practices and given the need for a tailored approach, each participant had developed an ad hoc system for managing incentives, but it was not always an easy task. They were not only responsible for distributing incentives but also keeping track of them (e.g., filling out paperwork), which at times made them feel like "bank tellers."

I said, "This is like ridiculous. It's too much." Like it's not money out of my pocket, but it was just dealing with, it was just something that I didn't want to deal with. I had enough to deal with. I'm a medical professional, I'm not a bank. (Participant 6)

Second, they were responsible for ensuring that the "right people" got incentives and following the rules. It could put them in a difficult position because care and service provision to marginalized communities often requires rule-bending. Bending the rules with incentives had different implications and was only done rarely—and only if seen as the right thing to do for the person.

We have a policy that we only give one meal because we have a limited number of meals to give to clients. But because you know this client really well and let's say they have just lost their home, and you know they've been sleeping under the bridge. And other clients have homes, so we end up sometimes making that kind of decision, "Okay, today we're giving this person a second meal to go." (Participant 11)

Third, they had to manage the relationship by using incentives in ways that fostered trust and engagement without being at odds with their professional values, standards, and goals. These included maintaining transparency, establishing clear boundaries, managing expectations, demonstrating flexibility, and being client-centred. However, the general feeling expressed by participants was that they were not prepared or equipped to work with the complexity of incentives or address the ethical tensions they may generate.

We identified several ethical tensions throughout the analysis. For this paper, we will focus on the most common: clarifying motives, obtaining free and informed consent, and balancing harms and benefits. The interviews revealed an interesting form of ethical reasoning. Participants wanted to make sure incentives were used for the "right" reasons, namely, for health, access, and care engagement purposes. Doing it for the "right" reasons made incentives feel more ethical. In this sense, rewarding people for doing something positive for their health, for their care and themselves felt better—and more aligned with professional goals and values.

I mean, sometimes we do buy people a snack and meals out of our own pocket because there's no money in the program to do that. And I think all of us kind of made that decision because we knew that this is the last dot that will connect us with the client who is very difficult to engage. You know what I mean? So, yeah, the reason I struggle with it is because I know, professionally, I'm not supposed to really spend my own money to buy something specifically for one client. And, we try to make sure that like people know that we're not doing this because we want a favour back from them. But it's really, really grey, I find. (Participant 11)

Participants felt conflicted about the prospect of giving out incentives for the "wrong" reasons (i.e., to get people to do things in return and to produce standardized results). In the interviews, there was a great deal of back and forth between expressing ethical discomforts and yet, hoping and believing that incentives could be used for the "right" reasons.

Like, I definitely have a reservation about providing incentives for people to do things. I have concerns about coercion under those circumstances. And I can understand that it may be in the client's best interest according to the health care provider's perception. So I do have concerns about that. And like, in my experience, I did sometimes feel kind of teetering on that edge of like, "Is this coercion, or is this ethical? Because I'm giving you something so that you do something, but it's not from your own free will. It's because you're receiving something from me." Like it's a transaction. And so, that I feel, there's something wrong there. Like in my, I feel in my body like there's something wrong with that sometimes. Not all the time. I feel that giving incentives to help facilitate someone's wellbeing and livelihood, and survival is completely different. (Participant 4)

Participants were aware of and concerned about the coercive power of incentives. At the same time, they were tasked with achieving specific targets for testing, treatment, and care and had to balance using incentives in ways that seemed ethically permissible to them.

And like, so ethically, sometimes like paying people to, like, it's basically, it's not a cash payment, but it is like a payment to get tested. It's kind of like ethically a little bit, like internally I'm kind of questioning like, "Should we? Should we be doing that?" But then also, it is providing connection to a service as well as a follow-up ability for that person because they do get something out of it. I think incentives are a useful tool at the end of the day. I think that if it's working for a population and we're getting results, then I think it is useful. I just wonder about, is it a slippery slope? (Participant 9)

The challenge of obtaining free and informed (explicit or implicit) consent when using incentives topped the list of ethical issues identified by participants. All of them described situations in which people just wanted to be "signed up," such as this one:

A lot of people who I reviewed the consenting process with didn't even want to hear what I had to say about it. They were just like, "Just sign me up. I just want the money." Which was quite problematic but, at the same time, quite understandable. (Participant 4)

As this participant pointed out, incentives influenced the consent process in ways that were problematic but understandable. Problematic because it made it nearly impossible to establish that participation in incentivized initiatives and programs was indeed voluntary—free of coercion or perceived coercion. Understandable, because incentives were seen as a source of survival income and could not be turned down. Participants struggled with this.

I don't know. I think that's one I continue to struggle with. And I don't, yeah, I'm not sure because I think the idea of true consent with incentives is really hard to achieve and pull out." (Participant 25, p. 12)

To mitigate this ethical tension, some participants had developed strategies including reviewing the details of what the incentivized initiative or program entailed before enrolling the person, assessing for readiness, watching for non-verbal cues that the person may not be comfortable, focusing on goal setting and trust-building, keeping lines of communication open and checking in with the person regularly, reminding the person that they do not have to participate, and so on.

As far as testing goes, if somebody, we do assess people to see for readiness of testing. And if they absolutely, like if they express things that make me concerned that they're not going to be safe if they were to have a positive HIV test, we wouldn't do the test. But I know that many people come in and just answer the questions and say, "It's fine," they want the testing so they can get the money. So that, that group of people does concern me a little bit, just in terms of people doing, the testing fully consenting and being prepared for an HIV test and HIV results. (Participant 10)

Despite using strategies, participants considered the consent process to be particularly difficult.

I thought, "So, we have these gift cards. It could be a tool more for engagement instead of coercion." I don't know. I think the ethics—and I'm glad that you're doing this research because I think it can get a little tricky with what's ethical and what's not. And if somebody is not ready to get in to do HIV testing, I don't think that—especially with the folks I work with, like a lot of them don't have secure housing or don't have food security. And so, kind of dangling a carrot in front of them, I don't know, I'm not, I'm not comfortable with that. (Participant 23)

Participants had many unanswered questions about the ethics of using incentives. They were unsure about what was ethical and what was not. They also found it challenging to determine what coercion might look like in the context of incentives. For example, was $5 enough to make someone take an HIV test they did not want to take that day? Was a cigarette or coffee enough to have someone take 10 min to talk to you when they have competing priorities? Was a free meal every day enough to have people take medications they did not want to take?

Again, to help mitigate these everyday ethical tensions, participants adopted an approach of balancing harms and benefits. Having no control over the design of incentivized initiatives and programs, they could at least try to maximize benefits and minimize potential harms. As such, they focused most of their efforts on the *relational* rather than the transactional aspects of their work. Incentives were seen through that lens—as tools for engagement and relationship building.

So, for clients that we had trouble engaging with all other methods, usually who were off their medications and just not accessing care, started coming, in which case we were able to develop rapport then and develop relationships. And people would, because they were safe in those relationships, would access care more and start thinking about medications and their health. (Participant 5)

However, they agreed that this approach came down to harm reduction. In other words, by working to keep the relationship feel authentic instead of transactional, they were trying to reduce the harms that come with using incentives into the context of service and care provision. The service and care provision had to change to achieve real, long-lasting, transformational changes. This is what participants were genuinely advocating for.

Yeah. It's really hard to figure out. Because on the one hand, it's like how sustainable is that to continue to have that transactional relationship? (…) Wouldn't it be better to build in like the deeper kind of like, I don't know, work with people around their health and health outcomes and what the barriers are? And kind of fund more providers to be able to have those conversations and ensure more doctors are trained and like increase like the amount of primary care providers and family doctors who can take patients and have, give them more time to have these conversations with folks? Those are the things I feel have more value than giving people money to take their medications. (Participant 25)

Overall, they considered that incentives—as a "Band-Aid" solution—fail to address structural barriers to care and structural determinants of health. Instead, they bring people at risk or living with HIV who experience the most inequities into a system that is not serving them. Using incentives was not going to fix this.

On the care side of things, it does seem like we're just, we're maintaining a status quo and offering people money to participate in a system that isn't really serving them, which is a shame. (Participant 10)

## Discussion

The main objective of this study was to document the experiences of health care and service providers with incentivized testing, treatment, and care in BC, Canada. Another aim was to explore the ethical and professional tensions that arise from this practice as well as strategies used by providers to mitigate them. Our findings suggest that participants did not implement incentives across the board, but instead selectively for target populations believed to pose the most risk to public health. As such, they used incentives to close the gaps in the HIV Cascade by getting the "right populations" to test, start treatment, stay on treatment, and, most importantly, achieve (and sustain) viral suppression. Incentives “worked” to some extent; they "brought people through the door" and "broke the ice" when engaging with clients who are hard-to-reach, hard-to-treat, and hard-to-retain in care. However, participants considered the effectiveness of incentives to be superficial, short-lived and one-dimensional—thus, failing to address underlying structural barriers to care and structural determinants of health. They also raised concerns about the unintended consequences of incentives on safety, inequity, risk, power dynamics, and engagement in care. Finally, our findings show that incentives came with added responsibility, such as managing interactions with clients and minimizing the potential strain on the therapeutic relationship. Participants had developed strategies to mitigate the ensuing ethical and professional tensions and to make their work feel relational rather than transactional.

Our findings locate the introduction of incentives at the heart of a large-scale medico-scientific and political project with a clear and ambitious goal: stopping HIV/AIDS by optimizing treatment as a tool for prevention for target—at-risk—populations [62, 63]. They raise several important issues worth noting here. The first issue is the convergence of a new way of conceptualizing HIV care—in the form of a linear cascade—with a new type of conditioning (i.e., incentivized care). Both focus on individual behaviours as the source of the problem and the primary site of intervention. Both are outcome-driven. Both detract attention away from the broader conditions shaping the experience of being at risk for or living with HIV. The second issue is the introduction of market logic into the realm of health care—an area of life previously not subject to monetary exchanges [64]. According to this logic, health interventions should be "utility-maximizing," meaning that they should be worth the time, energy, and commitment [65]. When the goal is to direct people to adopt particular health-related behaviours or to meet health outcomes, one way to maximize utility is to offer incentives in return [5]. As suggested in our findings, this logic is particularly effective when working with poor and marginalized communities because it draws on a familiar (transactional) frame of reference and boosts the utility of engaging care. These findings are consistent with the literature, which shows that incentives typically target low-income communities [3, 51]. Unsurprisingly then, and as highlighted in our findings, incentives usually target people who experience the most "budget constraints" [5]—a term used by behavioural economists. These constraints shape health care decisions in two ways: they can lead to disengagement in care by making it challenging to attend clinic appointments, for example, but with incentives, the same constraints can act favourably and increase the perceived utility-maximizing of engaging in care. To put it simply, if you need the money and the clinic gives you money to attend your appointments, you are more likely to attend. Finally, our findings revealed a third issue. During the analysis, it became clear that incentives were not simply about maximizing utility. If this were the case, they would not be contingent upon doing something: getting a test done, attending a clinic appointment, taking medications, achieving an undetectable viral load, and so forth. Our findings called for the need to think about incentives from a behaviour modification lens. Using that lens, we see similarities between our findings and those of scholars who have studied the ethics of behaviour modification programs (for example, see [66]). These programs, introduced after World War II, were the first to legitimize the use of a token system of punishment and reward to modify "deviant behaviours" [67]. We see, in the rise of incentives in HIV care, a similar legitimizing of behaviour control that calls for critical analysis.

This study indicates an urgent need to problematize the use of incentives as a part of the HIV Cascade agenda and to interrogate the ethics of engaging in this practice from the perspective of providers. As Seckinelgin [68] points out, the HIV Cascade is not designed to address the complexity of issues faced by people at risk and living with HIV—nor is it suited to inform policy and practice. Adopting this sequential model of care engagement driven by the goal of achieving sustained viral suppression and, more broadly, achieving public health goals of preventing HIV and cutting costs, shapes how providers intervene [68–72]. As such, our findings reaffirm the need to examine the ethical implications of using incentives against the backdrop of the HIV Cascade. In light of the experiences described by participants, and given the context in which they used incentives and the populations they used them with, we see significant ethical issues that merit further analysis in subsequent studies. We also see the need to engage with new ethical frameworks, grounded in relational and critical perspectives, as a complement to existing frameworks that rely heavily on classic principles of health care ethics [2, 3, 40, 42, 46, 47]. Interestingly, the existing literature suggests a tendency to use those principles to justify the need for incentives or to propose ways of using incentives "ethically" rather than engaging in an analysis of incentives per se. As such, we agree with Stephens [50] that a rigorous ethical analysis of incentives in health care starts with using frameworks that speak to the social and structural underpinning of health inequities and resist locating the responsibility for health at the level of individual behaviours. He argues that ignoring those fundamental aspects of health could increase social injustice and worsen health inequalities.

Our study points to deeper ethical issues that are difficult to mitigate by health care and service providers, especially those deeply committed to social justice and health equity. For example, incentives explicitly target people who experience the most health and social inequities and who are not in a position to decline the incentives—or what is being asked of them [51]. That is the primary motive for targeting them in the first place: "They are too poor to say no," as Voigt [51] puts it. Furthermore, they capitalize on existing micro-economies of survival by transforming health care relations and health care settings into sources of income (albeit a small income). From the perspective of providers, one must ask if this practice contributes to or ameliorates oppressive economic conditions [73]. We would argue that targeted incentives designed to be of the smallest value possible to yield results contribute to oppressive economic conditions [74]. Ameliorating these conditions would require a commitment to universal forms of supports such as housing, guaranteed minimum income, and food security. As pointed out by our participants, these forms of supports—or lack thereof—are powerful determinants of care engagement, medication adherence, and viral suppression [75–77]. Incentives shift the orientation of care away from such determinants and onto individual behaviours. As a result, the primary site of intervention becomes the individual who is attributed full responsibility for achieving sustained viral load suppression and bearing the downstream public health impact of their behaviours. Finally, there has been very little consideration given to the potential immediate and long-term harms of incentives [52] despite research showing their limited effectiveness [1, 22, 23]—including the long-term impact on caring relationships, engagement in care, and health outcomes. From an ethical standpoint, we argue that the potential harms of engaging in a practice that targets poor and marginalized populations, individualizes complex health issues, monetizes health care relationships, and contributes to oppressive economic conditions have the potential to outweigh incremental changes in metrics that are not sustainable over time.

We close this discussion by taking a closer look at the experiences of participants and the impact of incentives on the client-provider relationship. The majority of our participants had multiple experiences of using incentives in various roles. Overall, they reported mixed views about their experiences, in part because they saw incentives as having both positive (albeit limited) and negative effects and also because they were unclear about the ethical implications of using them in practice. Our findings are consistent with previous studies indicating that providers who use incentives report positive and negative effects on their care, relationships with clients, and health outcomes [14, 15, 78]. What is clear from our study and the literature is that using incentives in the context of a caring relationship raises many questions and concerns for providers—primarily related to power and autonomy. Tracing the history of incentives, Grant (2002) reminds us that they have always been "offered by people with power to people without it" [43]; first offered by employers to factory workers to enhance productivity (and profits) and now by providers to clients to optimize care engagement (and clinical outcomes). Grant [43] calls for incentives to be considered "a member of the set of ways in which power and influence are exercised—that is, as a form of control, rather than simply as an alternative to it." In the context of a caring relationship, this exercise of power and influence serves three separate yet complementary functions. First, it increases the effectiveness of incentives by giving them legitimacy as health care interventions. Second, it makes it more difficult for clients to say no, because of what this refusal could mean for their care or their relationships with providers. Third, it depoliticizes and neutralizes an economic intervention designed to modify behaviours considered expensive, risky, and unhealthy. In our study, providers used the concept of coercion to explain the nature of their questions and concerns. However, this concept fails to capture the subtle ways in which incentives mobilize power and influence. We argue that incentives are located somewhere between the "carrot" and the "stick" within a complex web of power relations, norms, and responsibilities that merit further analysis, especially in light of the HIV Cascade. In light of our findings, we see the need for further analysis of questions related to autonomy, power, vulnerability, and choice in incentivized care. The literature on research incentives can help tackle such questions, but as incentivized care continues to gain momentum, we also the importance of conducting more research and developing theoretical tools specific to the context of care. Finally, we believe that supporting providers who navigate a complex ethical terrain is paramount.

## Conclusion

This study offers a significant contribution to a small body of literature on the experience of health care and service providers tasked with administrating incentives in HIV Care in BC, Canada. We see four main strengths in this study: (1) richness of the data due to the range of experiences providers had accumulated across various roles, populations, and settings; (2) emphasis on studying hands-on experiences as opposed to acceptability, which has mostly focused on perceptions and attitudes to date; (3) inclusion of key informants capable of providing a broader context for the study including historical context; and (4) rigorous analysis including two-person validation of the matrix. However, some limitations should be considered when interpreting our findings. The study was based in the province of British Columbia and, as a result, may not reflect the reality of providers in other provinces or countries, especially in jurisdictions with higher rates of HIV infection and less access to health care. The majority of participants were nurses (*n* = 15), and as a result, they engaged in a discussion of their experience with incentives from a nursing standpoint and in light of their delineated scope of practice, professional roles and responsibilities, and ethical standards. Furthermore, it was limited to the care of people at risk or living with HIV, which may limit the transferability of the findings. Nevertheless, we found enough similarities in the literature to be confident that the findings are indeed transferable and relevant to the state of research on the topic. Finally, the study did not include the perspective of clients, and as such, it provides a one-sided view of incentives limited to the provider and not the recipient. It was also limited to the individual rather than the social. While participants did point to broader social issues to support some of their concerns about the use of incentives, the goal was to have them speak primarily from a place of experience and explore the use of incentives within a care context. For this reason, their analysis of this practice (and its impact) was grounded in individual and relational care more so than at a social level where benefits to society might come into play.

In light of our findings, we agree with Klein [3] that "the extent to which incentive programs are medical [or health] interventions rather than economic interventions is not clear." Because incentives are designed to be of the smallest amount possible for maximum effect and because they are targeted at hard-to-reach, hard-to-treat, and hard-to-retain populations who are typically poor and marginalized, we believe the latter to be more accurate. Economic interventions seek efficiency; they demand measurable results; and are more interested in conditioning than health. Still, in light of our findings, we agree with Klein [3] that "it is also not clear the extent to which incentive programs are medical [or health] interventions rather than public health interventions." This is an essential point because it speaks to underlying motives and benefits. Our study shows that incentives are consistent with the public health goal of reducing and eventually ending HIV transmission by ensuring that everyone who is HIV-positive is tested, treated, retained in care, and virally suppressed. However, as pointed out by Seckinelgin [68], people who are at risk or living with HIV "do not live in the cascade." Many of them live busy, chaotic, and complex day-to-day lives with a focus on survival. What does health look like for them? What matters to them? Beyond the virus. Providing ethical care in the era of the HIV cascade starts by shifting from "care of the viral to care of the vital" [71]. Regardless of whether the context is well resourced or considered ‘developing’ with a high burden of disease, we argue that incentives should only be considered following a process of consultation where the incentives have been deemed meaningful by recipients, and should only be used alongside efforts to promote health equity, social justice, and systemic change.

## Data Availability

In order to protect the confidentiality and anonymity of participants, the data (transcripts) will not be shared.

## Abbreviations

AIDS: Acquired Immunodeficiency Syndrome
ATA: Applied Thematic Analysis
BC: British Columbia
CAD: Canadian Dollars
DTES: Downtown Eastside
HIV: Human Immunodeficiency Virus
STOP HIV/AIDS®: Seek and Treat for Optimal Prevention of HIV/AIDS program
STI: Sexually Transmitted Infections

## Footnotes

1 https://cfenet.ubc.ca/tasp

2 https://stophivaids.ca/

3 Patients demonstrating the identified target behaviour earn draws from a fishbowl in a systematic manner, either in a set number or with an escalating schedule. Probability is introduced via an intermittent reinforcement schedule in which some draws are winning (“small prize”) and others have no prize value (“good job”). A typical fishbowl is comprised of 500 slips, about half of which are winning. Prize earnings indicated on winning slips vary in magnitude from $1 to $100, with the majority of winning slips comprised of the smallest magnitude ($1). A smaller number provide large magnitude (e.g., $20) prizes, and one slip in the bowl is the maximum prize ($100) [61].
